# Microplastics induce transcriptional changes, immune response and behavioral alterations in adult zebrafish

**DOI:** 10.1038/s41598-019-52292-5

**Published:** 2019-10-31

**Authors:** Giacomo Limonta, Annalaura Mancia, Assja Benkhalqui, Cristiano Bertolucci, Luigi Abelli, Maria Cristina Fossi, Cristina Panti

**Affiliations:** 1 Department of Physical, Earth and Environmental Sciences, University of Siena, Siena, 53100 Italy; 2 Department of Life Sciences and Biotechnology, University of Ferrara, Ferrara, 44121 Italy

**Keywords:** Ecology, Environmental impact

## Abstract

Microplastics have become pervasive environmental pollutants in both freshwater and marine ecosystems. The presence of microplastics have been recorded in the tissues of many wild fish species, and laboratory studies have demonstrated that microplastics can exert adverse health effects. To further investigate the biological mechanisms underlying microplastics toxicity we applied an integrated approach, analyzing the effects of microplastics at transcriptomic, histological and behavioral level. Adult zebrafish have been exposed to two concentrations of high-density polyethylene and polystyrene microplastics for twenty days. Transcriptomic results indicate alterations in the expression of immune system genes and the down-regulation of genes correlated with epithelium integrity and lipid metabolism. The transcriptomic findings are supported by tissue alterations and higher occurrence of neutrophils observed in gills and intestinal epithelium. Even the daily rhythm of activity of zebrafish appears to be affected, although the regular pattern of activity is recovered over time. Considering the transcriptomic and histological findings reported, we hypothesize that the effects on mucosal epithelium integrity and immune response could potentially reduce the organism defense against pathogens, and lead to a different utilization of energy stores.

## Introduction

Nowadays, marine and freshwater environments all around the world are subject to plastic pollution, which is affecting every compartment of the aquatic ecosystems, from the surface waters to the sediments^1–4^. Moreover, under the action of natural factors, such as photodegradation and wave action, plastics litter is gradually broken down into particles smaller than 5 mm, defined as microplastics (MPs)^5,6^. As the issue of MPs pollution has become of rising concern, an increasing number of studies have investigated their effects on biota. The effects of MPs on aquatic organisms can be roughly divided into two categories: i) chemical, due to the transfer of environmental contaminants adsorbed on the MPs surface or the leaching of plastic monomers/additives, and ii) physical, due to damage caused by the direct interaction of the plastic particles with the organism tissues^7–9^. It has not been completely elucidated which one of the two categories of effects pose the bigger threat and is the most responsible for MPs toxicity. However, some studies have questioned the relevance of MPs as a pathway for the transfer of environmental contaminants to the biota, suggesting that natural agents such as water, food, and organic particles, may play a more predominant role^10,11^. With respect to the polymeric composition, polyethylene (PE), which is the polymer produced in the largest quantities worldwide^12^, represents the most abundant constituent of marine and freshwater MPs pollution^4,13,14^. Polystyrene (PS) is slightly less abundant than PE in the environment, but it has been suggested that its aromatic monomer, styrene, could cause acute toxicity and endocrine disruption^15,16^. MPs can be easily taken up by fish, and have been isolated from the gastrointestinal tract (GIT), gills, liver and muscle of wild specimens^17–19^. Laboratory studies suggest that the two main routes of uptake in fish are represented by the GIT and gills, where MPs can accumulate and cause tissue damage^9,20,21^. In addition, the presence of MPs have been recorded in the liver of zebrafish experimentally exposed to PS MPs, together with the induction of anti-oxidative enzymes activity and the alteration of metabolites composition^21^. Overall, histological examinations on exposed fish confirmed that MPs were able to induce a strong inflammatory response in the target tissues^9,21,22^. The transcriptomic profiling of zebrafish larvae exposed to PS MPs also suggested the activation of the immune response, with the up-regulation of genes related to the complement system^23^. However, a comprehensive dataset on the molecular pathways affected by MPs exposure in adult fish is still lacking. In addition, only a few data are available about the behavioral effects of MPs in fish, although it has been reported the inhibition of acetylcholinesterase activity^24^, which is essential for synaptic neurotransmission. The objective of this study was to enhance the current knowledge regarding the effects of MPs on aquatic organisms, investigating which biological processes and pathways are affected, using a combined transcriptomic, histopathological and behavioral approach. We focused on the effects of virgin plastic particles, exposing adult zebrafish (*Danio rerio*) to different concentrations of a mixture of high-density polyethylene (HD-PE) and PS MPs. Zebrafish was selected as the most suitable experimental organism, considering the advantages of working with a deeply known vertebrate species with an entirely sequenced genome^25^.

## Results

### Effects on the liver transcriptome

**Figure 1:**
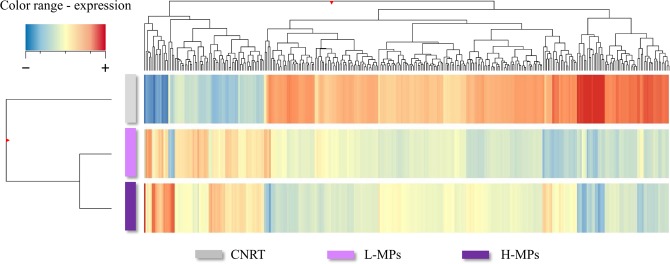
Hierarchical cluster of the differentially expressed genes across treatments. Genes and samples are clustered by similarity of expression (top and left, respectively). CNTR (control); L-MPs (100 µg/L); H-MPs (1000 µg/L).

**Figure 2:**
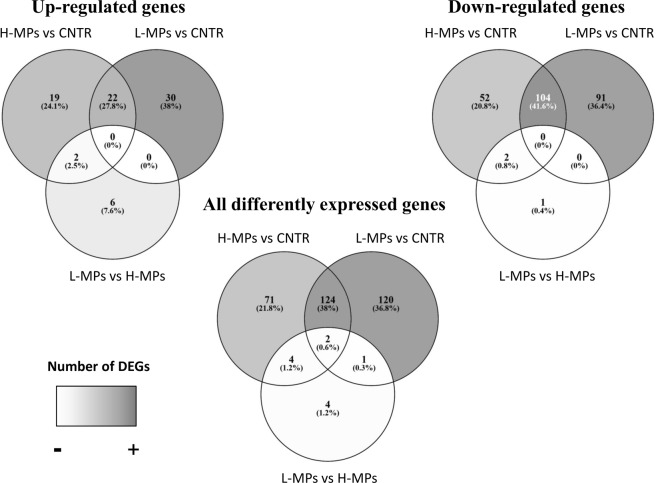
Venn diagrams showing the number of DEGs after 20 days of MPs exposure. The number of all DEGs, up-regulated genes and down-regulated genes are shown for every comparison: H-MPs (1000 µg/L) vs CNTR (Control), L-MPs (100 µg/L) vs CNTR (Control) and L-MPs (100 µg/L) vs H-MPs (1000 µg/L).

The RNA sequencing performed on the liver samples generated approximately 53 million reads per sample and a total of 25357 unigenes were individuated. On average, the GC content of the sequences generated was 48%, while the sequence quality score was above Q30 for 93,81% of the reads (Table 1). The heatmap and hierarchical clustering performed on the DEGs highlight the substantial differences between experimental groups following MPs exposures (Fig. 1). A total of 326 gene transcripts were found differentially expressed across conditions (Fig. 2). Of these, 126 were in common between the two MPs treatments, 121 we differently expressed exclusively after L-MPs treatment (100 µg/L MPs), and 75 exclusively after H-MPs (1000 µg/L MPs) (Fig. 2). Only 4 genes were found significantly differentially expressed between the two MPs loads (Fig. 2). A complete list of the DEGs across all conditions, with fold change of expression and statistical significance (*p*-value and FDR), can be found in Supplementary information Table S10.

**Table 1:** Sequencing metrics and quality check results for RNAseq raw reads.

Sample name	Raw reads	Q30	Mapped reads	GC content
CNTR_1	43,574,088	94,08%	22,449,125 (51,52%)	48%
CNTR_2	59,758,664	93,86%	37,481,952 (62,72%)	47%
L-MPs_1	55,592,971	94,12%	24,939,160 (44,86%)	49%
L-MPs_ 2	56,170,923	93,38%	20,255,669 (36,06%)	50%
L-MPs_ 3	51,710,923	93,45%	28,224,903 (54,58%)	48%
H-MPs_1	61,550,230	93,83%	32,369,773 (52,59%)	47%
H-MPs_ 2	49,936,176	93,68%	24,632,151 (49,33%)	48%
H-MPs_ 3	51,985,614	94,10%	28,323,164 (54,48%)	48%

Control samples (CNRT), 100 µg/L of MPs (L-MPs) and 1000 µg/L MPs (H-MPs). Q30 value represents the percentage of reads above quality score 30.

**Figure 3:**
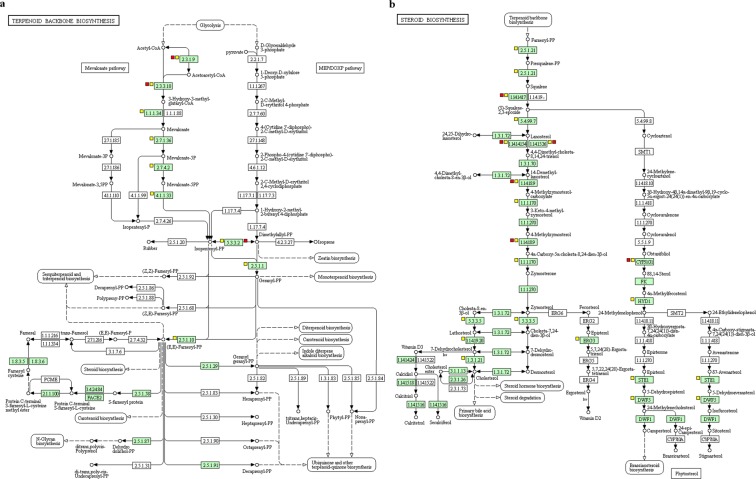
KEGG pathways involved in the lipid methabolism of zebrafish enriched after MPs exposure. (**a**) Terpenoid backbone biosynthesis (L-MPs FDR = 2.40E-07; H-MPs FDR = 0.191679). (**b**) Steroid biosynthesis (L-MPs FDR = 1.11E-08; H-MPs FDR = 0.228282). Green boxes represent genes involved in the organism-specific pathway for *Danio rerio*. Yellow squares next to the pathway steps indicate genes that are down-regulated transcribed after L-MPs exposure (100 µg/L). Red squares next to the pathway steps indicate genes that are down-regulated transcribed after H-MPs exposure (1000 µg/L). Pathway images obtained from KEGG^50,51^.

The output of the GO analysis performed with DAVID online found 10 enriched GO terms in the H-MPs group (*p* < *0.05*) and 28 for the L-MPs group (*p* < *0.05*), in comparison with the control. Most of the enriched terms in either treatment were related to the lipid metabolism, such as sterol biosynthetic process (GO:0016126), steroid metabolic process (GO:0008202) and fatty acid metabolic process (GO.0006633) (Supplementary Tables S3 and S4). Consistently with the GO results, the pathway analysis carried out on DAVID online using the KEGG pathways available for zebrafish, found the most striking overlap with the steroid biosynthesis pathway (dre00100) and the terpenoid backbone biosynthesis (dre00900), which were affected by either MPs treatments (Fig. 3, Supplementary Tables S5 and S6). The steroid biosynthesis pathway is represented in zebrafish by the activity of 21 genes in total, 3 of which were found down-regulated after the H-MPs treatment, 9 of which were down-regulated after the L-MPs treatment. The terpenoid backbone biosynthesis comprises 22 genes in total, 3 of which were found down-regulated after the H-MPs treatment, 8 were down-regulated after the L-MPs treatment (Fig. 3). For all the GO terms (Supplementary Tables S3 and S4) and KEGG pathways (Supplementary Tables S5 and S6), a higher fold change of enrichment and statistical significance (FDR < *0.05*) was found in the L-MPs treatment.

### Validation of RNAseq data through qRT-PCR

The results of the qRT-PCR performed on the selected genes showed an evident correlation of fold change of expression with the RNAseq results (Supplementary Fig. S5). The genes were selected on the basis of statistical significance (adjusted *p*-value/fold change) returned by the DEGs analysis performed with DESeq2 and functional diversity: immune response (*ltb4r, ifitm1*), lipid metabolic process (*elovl6, ch25h*), oxidation-reduction process (*cyp51*). A slightly different magnitude of fold change has been observed between qRT-PCR and RNAseq results, this discrepancy has been observed in previous studies^26^ and can be attributed to intrinsic differences between the two techniques.

### Histological findings

#### Gastrointestinal tract

**Figure 4:**
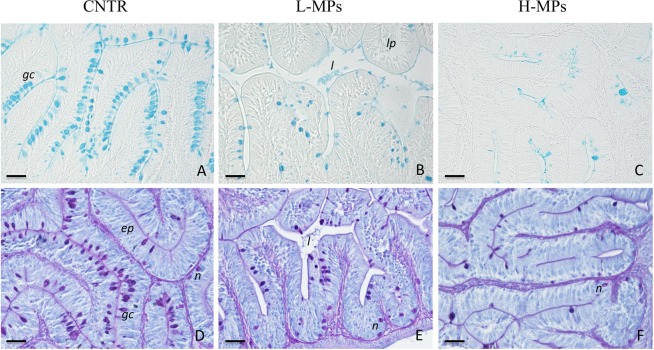
Histology of gastrointestinal tract (GIT). Representative sections of intestines stained with AB (**A–C**) or PAS with HH nuclear counterstain (**D–F**). CNTR (Control) in (**A,D**); L-MPs (100 µg/L) in (**B** and **E**); H-MPs (1000 µg/L) in (**C,F**). *Gc*: goblet cells; *l*: lumen; *lp*: lamina propria; ep: epithelium; n: neutrophils. Scale bar: 50 µm.

Zebrafish exposed to MPs showed alterations of the intestinal mucosa, such as epithelial detachment and mucous hypersecretion (Fig. 4). Significant differences (*p* < 0.05) in the occurrence of goblet cells and neutrophils were detected between MPs exposed groups and control (Table 2). In particular, the PAS and AB stains detected much fewer goblet cells than in controls (Fig. 4; Table 2). PAS reaction allowed mucosal neutrophils to be detected and quantified at higher density in MPs-exposed fish (Fig. 4D–F; Table 2).

**Table 2:** Summary of quantitative data yielded from histological analyses.

	Histopatological score		Intestine (N cells/mm^2^)		Intestine (N cells/mm^2^)	Gills (N cells/mm^2^)
			Goblet cells		Neutrophils	Neutrophils
	GI tract	Gills	PAS^+^	AB^+^	PAS^+^	PAS^+^
CNTR	0.3 ± 0.6	0.3 ± 0.3	58 ± 22	99 ± 4	10 ± 1	1 ± 1
L-MPs	1.2 ± 0.8	1.2 ± 0.3*	35 ± 13	55 ± 38*	21 ± 4*	11 ± 8*
H-MPs	1.5 ± 0.5	1.7 ± 0.3*	31 ± 24	52 ± 31*	18 ± 12*	9 ± 6*

The histopathological score was graded as 0: healthy tissues, 1: mild alteration, 2: gross alteration. Results are expressed as the mean ± SD (N = 3 specimens per group). *Significantly different from control *p* < 0.05.

#### Gills

**Figure 5:**
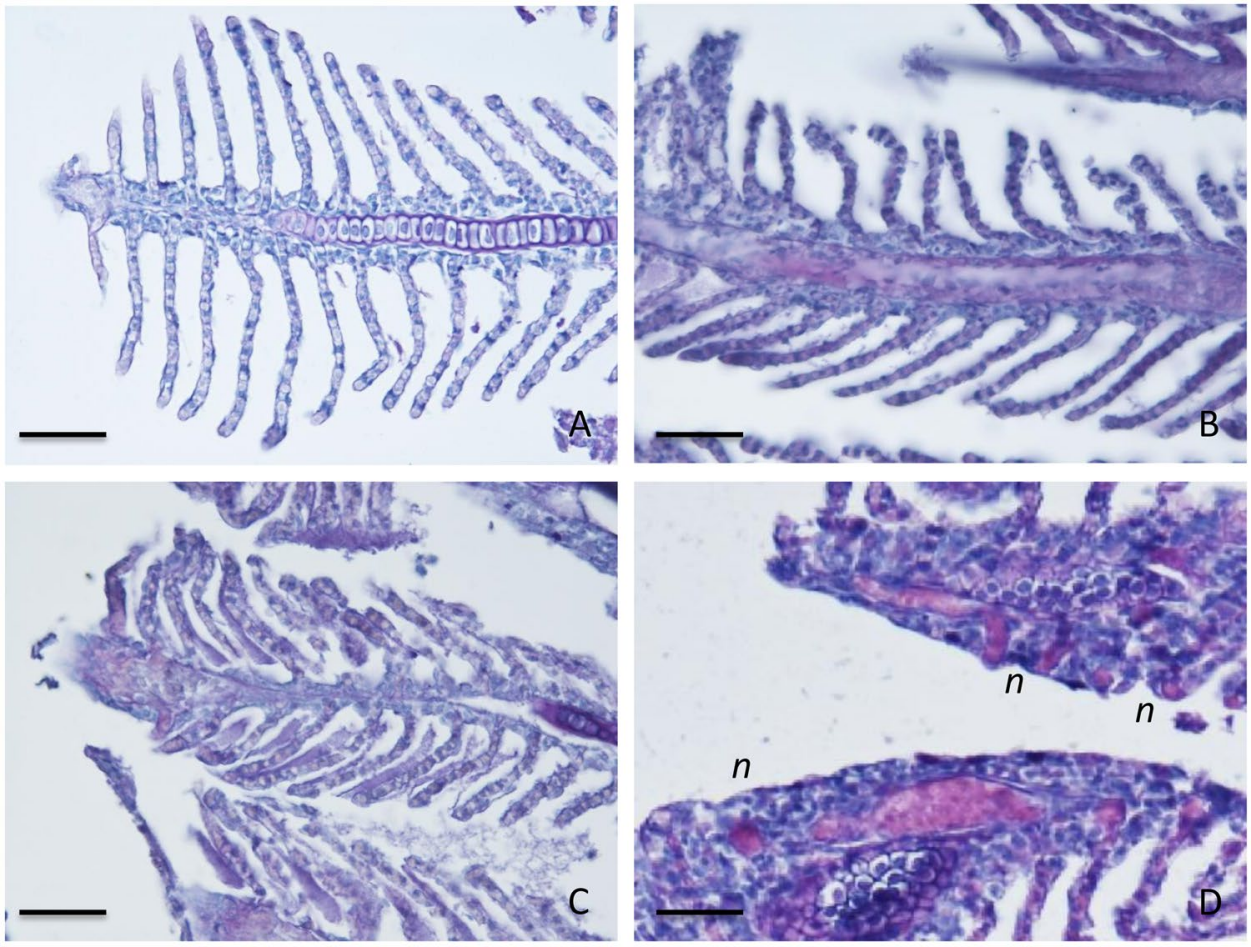
Histology of gills. Representative sections of gill lamellae stained with PAS with HH nuclear counterstain. CNTR (Control) in (**A**); L-MPs (100 µg/L) in (**B**); H-MPs (1000 µg/L) in (**C**). Note in (**D**) (L-MPs) high occurrence of neutrophils (n). Scale bar: 50 µm.

Zebrafish exposed to MPs also showed alterations of gill epithelium, such as adhesion and partial fusion of secondary lamellae and mucous hypersecretion (Fig. 5B,C). Similarly to what has been observed for the GIT, significant differences (*p* < 0.05) in the histopathologicl score and neutrophils occurrence were detected between MPs exposed groups and control (Table 2). With the aid of PAS reaction, a higher density of neutrophils was recorded and quantified in gill tissue of MP-exposed fish (Fig. 5; Table 2).

### Activity recording results

**Figure 6:**
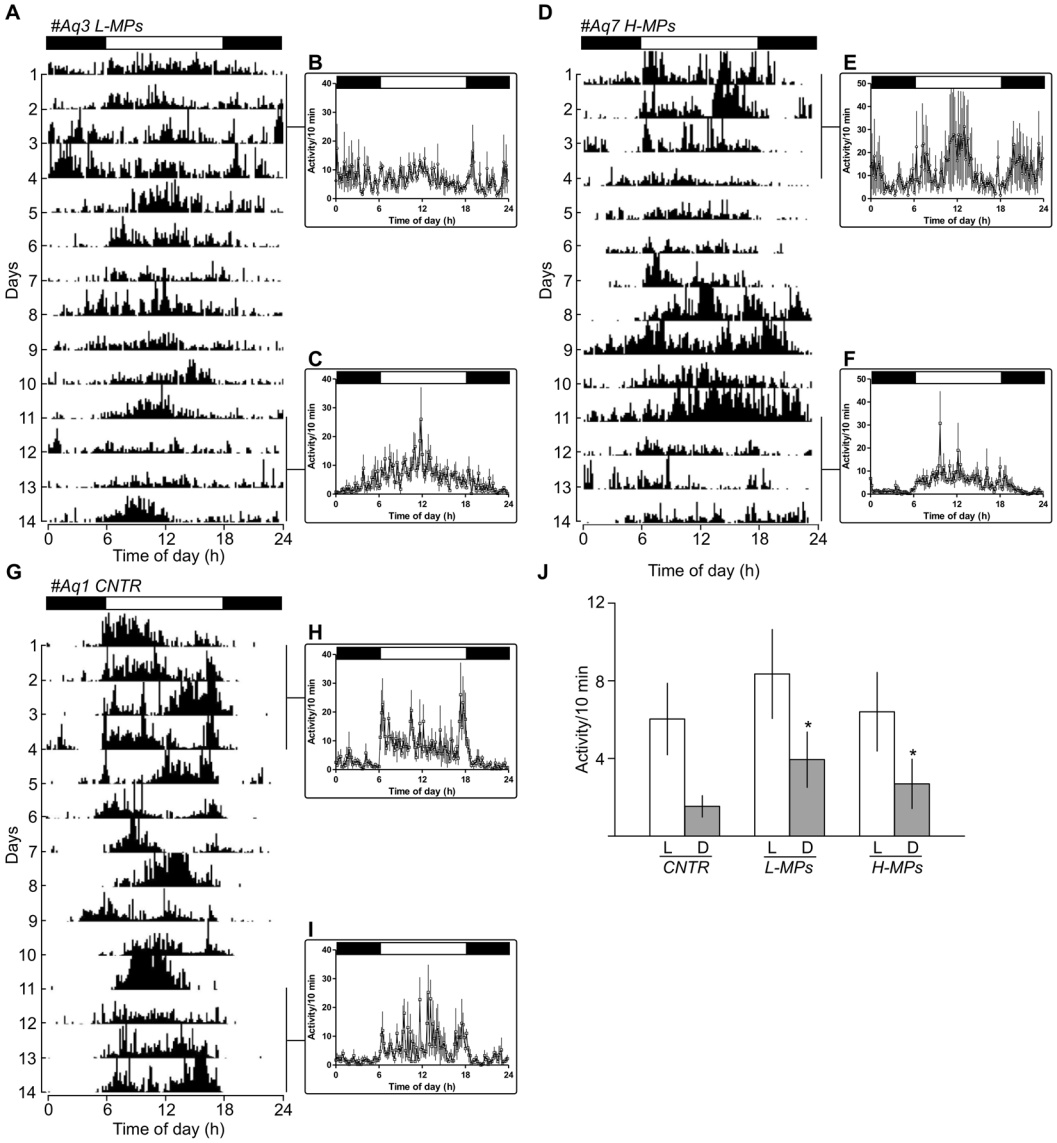
The daily activity rhythm of zebrafish is affected by MPs. Representative actograms (**A,D,G**) and mean waveforms of locomotor activity (**B,C,E,F,H,I**) in zebrafish housed under a 12:12 LD cycle. The white and black bars at the top of the graphs indicate the light and dark periods, respectively. In actograms the height of each point represents the number of infrared light-beam interruptions per 10 min. In mean waveforms, each point was calculated as the mean ± SD from the 10-minute binned data across 4 experimental days. (**J**) Amount of activity (mean ± SD) measured during photophases (white bars) and scotophases (gray bars) in the different experimental conditions. *p < 0.05

The daily activity rhythm of zebrafish is affected by MPs. As expected control zebrafish showed a clear daily rhythm of locomotor activity synchronized to the 12:12 LD cycle (Representative example in Fig. 6G), with higher activity (>75%) during the light phase (Fig. 6H–I). Differently, fish exposed to MPs showed an altered pattern of activity (Representative example in Fig. 6A,D). Particularly, the nocturnal activity of zebrafish exposed to L-MPs or H-MPs (Fig. 6A–F) was significantly higher (+10% and +20%, respectively; K8 = 339, p < 0.0001) respect to the control (Fig. 6G–I). The increase of nocturnal activity was showed during the whole recording (Fig. 6J).

## Discussion

This study aimed to elucidate the potential effects of HD-PE and PS MPs in adult zebrafish analyzing the responses to MPs exposure at transcriptomic, histological and behavioral level.

Most of the changes recorded in the liver transcriptome were already activated by exposing adult zebrafish for 20 days to the lower MPs concentration (100 µg/L), which is comparable to concentrations measured in highly polluted marine and freshwater environments^27,28^. Although it is not possible to deduce dose/effect relationship with the data here presented, which was not the aim of the study, we report that the transcriptomic alterations observed after exposure to the lower MPs concentration (100 µg/L) are substantially overlapped by those exerted by the higher concentration of MPs (1000 µg/L). The main effects on gene transcription were attributed to i) immune enhancement *versus* extracellular antigens and depression *vs*. intracellular ones, and ii) down-regulation of energy metabolism pathways.

In total, 41 differentially expressed genes (17 up- and 26 down-regulated) were attributed to immune response, most of them were differentially transcribed after both MPs treatments (Supplementary Table S7). Up-regulation was recorded for key role genes subserving MHCII processing and antigen presentation (*cd74a*, *cd74b*, *ciita*), lymphocyte activation (*ccr7*, *ccl19a*, *ccl19b*), innate immunity (*trim-35-23*, *trim35-24*, *trim35-25*, *chia.3*) and general activation of the immune response (*ccl38.6, lym6m3, tcirg1a, tcimb*) or cell proliferation (*mia, lect2l*). Down-regulation was found for gene transcripts involved in innate antimicrobial response (*marco, hsp60, hsp70*), antiviral defense (*ifit8*, *ifitm1*, *batf3*), ROS formation (*nox5*, *noxo1a*, *slc7a11*), angiogenesis, cell adhesion and maintaining of cell junctions (*lpar6a*, *fn1a*, *creld1b*, *cscl8a*, *cers2b*, *mmp14a*, *mmp14b*, *alox5b*). It is worth noting the down-regulation of *claudin b (cldnb)* e *crumbs homolog 3b* (*crb3b*) transcripts, due to the relevant role played by these gene products for epithelium integrity. The modulation of the expression for genes related to oxidative stress response and immune processes following MPs exposure have been previously documented^29^, although the molecular and biological mechanisms activated, remain to be fully elucidated. For instance, according to transcriptomic analysis performed on *Pocillipora damicornis*, MPs were able to alter the JNK and ERK pathways, possibly undermining the immune response^30^. In mussel, MPs affected the expression of important immunity genes of the PRRs and AMPs family, which have a central role in the immune functions of bivalves^31^. Fewer transcriptomic data are available for fish, but a recent study conducted on zebrafish larvae reported that PS MPs can lead to the alteration of complement system genes^23^. According to the transcriptional profile that we observed, the down-regulation of innate immunity and epithelial integrity genes, suggest that MPs can affect fish immune functions, with possible defeated control of pathogen entry at epithelial barriers and rising chances of infections at mucosal sites.

The alteration of the liver transcriptome is coherent with the histopathological signs detected in both GIT and gills epithelium. The lower number of detectable goblet cells in the GIT epithelium could be in part interpreted as due to mucous discharge, but is particularly relevant to consider that AB + mucus has been linked to the higher occurrence of anti-microbial peptides, thus indicating impairment of a relevant first-line defense against pathogen penetration. Enhanced expression of immune genes involved in response against extracellular antigens (eg. MHCII, and markers of neutrophils and APC) is outlined by overt mucosal neutrophilia and it is coherent with an excessive entry of exogenous antigens. Zebrafish exposed to MPs also showed alterations of gill epithelium, enhanced occurrence of neutrophils, adhesion and partial fusion of secondary lamellae and mucous hypersecretion. The reduction of epithelial integrity observed is consistent with previous studies, which reported the capacity of MPs to induce epithelial damage in the GIT and gills tissue, such as epithelial detachment and loss of gills secondary lamellae^32^. Interestingly, another study comprising zebrafish exposure to 1000 µg/L of PS MP, reported the augmented mucous secretion in the GIT, followed by the increase of pathogenic bacteria in the gut microbiota^33^. The capacity of MPs to alter the gut microbiota in favor of pathogens could represent an additional factor correlated with the transcriptional modulation of the immune response that was observed in the present study.

A cascade effect of the impaired health appears represented by the shift in the energy metabolic pathways, although we did not observe evident changes in food consumption throughout the experiment. Among the DEGs, 23 genes were attributed to lipid metabolism, all of them were down-regulated after MPs exposure (Supplementary Table S8). In particular, the lipid metabolism is altered by the down-regulation of genes involved in either steroid/sterol metabolic processes (GO:0008202, GO:0016126) and fatty acid biosynthetic process (GO:0006633). Genes that transcribe for transmembrane proteins responsible for fat storage and lipid droplet formation (*fitm2*, *gpr84*) were down-regulated as well^34,35^. Accordingly, the terpenoid backbone biosynthesis and the steroid biosynthesis pathways were the most impacted, These two are linked pathways that, within the sterol biosynthesis pathway, lead to the production of cholesterol. The down-regulation of many key genes within the mentioned pathways (*sqlea, ch25h, msmo1, mvda, cyp51, dhcr7 hmgcs1, hmgcra*) after both MPs treatments, strongly suggest an inhibitory effect on cholesterol production (Fig. 3). The down-regulation of sterol-related genes may also be linked to the down-regulation of *the sterol regulatory element binding transcription factor 2* (*srebf2*), which is known to regulate their synthesis^36^. Although plasmatic cholesterol level was not measured in this study, previous studies have reported the capability of MPs to disrupt energy and lipid metabolism^21,32^. The reduction of blood and plasma cholesterol levels was reported following MPs exposure in African catfish (*Clarias gariepinus*)^32^, as well as changes in the composition of triglycerides and fatty acids metabolites in zebrafish liver exposed to µPS^21^. Even in mice, dietary exposure to PS MPs caused the decrease of hepatic triglyceride and total cholesterol levels, coupled with the down-regulation of genes involved in lipogenesis^33^. The exact mechanism by which the lipid metabolism is affected by MPs exposure needs to be further investigated, it has been hypothesized that reduced food adsorption due to MPs interference in the diet may be the cause^21^. However, the role played by sterols and other lipids in inflammation and its interaction with immune system components^37^ suggest that there may be a link between metabolic alteration, inflammation and immune responses. For instance the deficiency of *long-chain fatty-acid elongase* (e*lovl6*) in mice was correlated with increased skin inflammation and keratinocyte death following mechanical damage^38^. On the other hand, cholesterol is essential for many cellular functions, it is transferred from the mother to the embryo in early developmental stages of vertebrates^39,40^, and it is required for correct development. Pharmacological inhibition of cholesterol production in adult zebrafish down-regulated some of the genes whose expression was inhibited by MPs as well (*cyp51, dhcr7*, and *hmgcra*). In that case, the inhibition of *cyp51, dhcr7*, and *hmgcra* by statin treatment ultimately lead to the disruption of embryonic development in zebrafish offspring^41^, raising questions on the potential effects of parental exposure to MPs on zebrafish larvae and embryos health.

MPs exposure up-regulated *tcim* gene (promoter of cell proliferation, apoptosis inhibitor, involved in thyroid and lung cancer in humans, and down-regulated *cers2b* (encoding in humans *tumor metastasis-suppressor gene 1* protein, liver regeneration promoter), *tp53inp1* (p53 inhibitor implicated in cancer progression), *agr2* (involved in cell migration, transformation and metastasis) and *wwox* (tumor suppressor that plays a role in apoptosis) genes (Supplementary Table S9). This gene expression profile suggests that there may be a predisposition of hepatocytes to undergo transformation and give rise to tumor development, which is consistent with the findings presented by Rochman *et al*., which observed the presence of a tumor precursor in the liver of Japanese medaka (*Oryzias latipes*), following exposure to virgin LD-PE fragments^8^.

Even the daily rhythm of locomotor activity was altered by MPs exposure. Zebrafish exposed to the MPs activity had a significant increase in the activity during the dark phase determining the loss of the regular diurnal pattern of activity. Interestingly, a recent investigation^42^ showed that plastic nanoparticles were able to alter feeding behavior in Crucian carp (*Carassius carassius*). In that case, the nanoplastics were able to affect fish behavior crossing the blood-brain barrier, which we believe it is unlikely to be happening in the present experiment considering the dimension of the particles used. The cause of the increased activity induced by MPs during the dark phase is not clear, however, it is worth mentioning that the up-regulation of the circadian clock gene *nr1d4b* in liver transcripts was observed in experiment I. Although it is not possible to directly correlate the RNA sequencing and the behavioral data, as results of two independent experiments, the data obtained suggest a possible alteration on the circadian timekeeping mechanism, nevertheless, further studies on the subject are needed.

In conclusion, we report that exposure of adult zebrafish to environmentally relevant concentrations of a mixture of HD-PE and PS can alter the expression of genes related to immunity and metabolic pathways in the liver, affect the tissue integrity of GIT and gills, where higher occurrence of leucocytes was detected, and have also an impact on zebrafish behavior. Although we did not study the fish response to a concurrent MPs and pathogen exposure, we hypothesize that the reported effects could lead to fading of innate immune mechanisms and first-barriers against pathogen penetration, which could bring the organism to responses implying a different utilization of energy stores. To confirm this hypothesis further studies comprising MPs exposure with a concurrent pathogen challenge are needed. Overall, this study contributes to filling the still existing knowledge gap on the mechanisms of MPs toxicity on aquatic organisms at different biological levels.

## Methods

### Experimental design

Adult zebrafish were housed according to standard method^43^ (14:10 LD cycle and 26 ± 1 °C of temperature) and acclimated in 30 L glass tanks for 2 weeks before the experiment. All husbandry and experimental procedures complied with European Legislation for the Protection of Animals used for Scientific Purposes (Directive 2010/63/EU) and were previously authorized by the University of Ferrara Institutional Animal Care and Use Committee and by the Italian Ministry of Health (auth. num. 702/2017-PR).

Virgin HD-PE and PS used were irregularly shaped particles with the following size distribution: 90% < 90 µm; 50% < 50 µm; 10% < 25 µm (Toxemerge Pty Ltd, Australia). The polymeric composition of the virgin MPs was confirmed using Fourier Transform Infrared Spectroscopy (FTIR). The analysis was performed using a Cary 630 FTIR spectrometer (Agilent), the standard spectra for HD-PE and PS present in the instrument library (Agilent Polymer ATR Library) were used as a reference for the comparison (Supplementary Fig. S1).

Two consecutive experiments were performed: the first one aimed at evaluating variations of the liver transcriptome and alterations of GIT and gills histology following MPs treatment, while the second one evaluated behavioral responses, specifically focusing on fish locomotor activity. The MPs concentrations, the method, and duration of the exposure used were the same for both experiments. Zebrafish were exposed for 20 days to two different concentrations (100 and 1000 µg/L) of a mix of MPs (50% HDPE and 50% PS). Zebrafish were fed daily at 2% of the average body weight of all specimens (Supplementary Table S1) with experimental food prepared mixing commercial dry food (Tetra, Spectrum Brands Company, Italy) with the MPs mix. Each day before feeding the water was fully replaced and the tanks rinsed thoroughly to avoid MPs accumulation. Air stones placed in the tanks provided water oxygenation and movement, thus maintaining the particles in suspension and reducing uneven dispersion and surface interactions among particles or with the tank walls^44^. Visual inspections confirmed almost complete food consumption within 30 minutes.

### Experiment I: Transcriptome profiling and histopathological examinations

Adult zebrafish specimens (n = 4 per tank; n = 36 in total) were placed in 9 tanks, each containing 1 L of water. Three replicate tanks were assigned to each experimental condition: Control (C), 100 µg/L MPs (L), 1000 µg/L MPs (H). Zebrafish were exposed to MPs for 20 days following the protocol beforehand described. At the end of the exposure, all animals were euthanized by anesthetic overdose (200 mg/L tricaine methane-sulfonate, MS-222) prior to tissues sampling.

### Tissues sampling

Zebrafish gender, total length, body, and organ weight were recorded (Supplementary Table S1), liver, spleen, GIT, gills and head kidney were surgically dissected and collected. Three out of 4 zebrafish from each tank were randomly selected for transcriptomic analysis, while the fourth one was sampled for histological analysis. For transcriptomic analysis (RNA-sequencing and quantitative real-time PCR), the liver was immediately submerged in RNA*later* (Sigma-Aldrich, Missouri, USA) and stored at −20 °C until RNA extraction. For histology, the GIT and the gills were fixed in Bouin’s liquid for 7 hrs at 4 °C.

### Processing and analysis of histological samples

GIT and the gills samples were dehydrated in graded cold ethanol series and embedded in paraffin wax. Serial sections (6 µm thick) were stained with Harris’ haematoxylin (HH) and eosin (E) for general histology, or with Alcian blue (AB, pH 2) or periodic acid-Schiff (PAS) for acidic mucopolysaccharides and carbohydrates, respectively. In the latter nuclei were counterstained by HH. The staining of carbohydrates was controlled by preincubating adjacent sections for 25 min at 37 °C with α-amylase before PAS or by the omission of the oxidation with periodic acid. To quantify the histological alterations, the histopathological score was graded as follows, 0: healthy tissues, 1: mild alteration, 2: gross alteration, based on leucocyte occurrence in intestinal mucosa or gill lamellae, neutrophilia, epithelial integrity and layering, and mucous storage and secretion. The occurrence of neutrophils and goblet cells was quantified as number of cells per mm^2^ of tissue.

### Total RNA isolation

Liver samples from three fish of the same tank were pooled, obtaining a total of nine samples (e.g. 3 biological replicates for each experimental group). Pooling was chosen because of the small tissue sizes and aiming at reducing inter-individual variability. Total RNA was extracted using RNeasy Plus Mini Kit (Qiagen, Hilden, Germany) following the manufacturer’s instructions. Tissue lysis and homogenization was performed with a TissueLyser (Qiagen). Genomic DNA was removed through an in-column DNase I digestion (Qiagen). The RNA concentration and the quality of the extractions were firstly assessed with a Nano-Drop ND-100 UV–Vis spectrophotometer (NanoDrop Technologies LLC, Wilmington, USA) and then with a 2100 Bioanalyzer (Agilent Technologies, Santa Clara, USA). The RNA samples showed good 280/260 nm and 260/230 nm ratios and high RNA integrity number (RIN): 8.92 average (range 8.3–9.2). Except for one pool from the C group that did not meet the QC standards (RIN) and was excluded from further analysis, leaving a total of eight samples: 2 CNTR, 3 L-MPs and 3 H-MPs (each comprising 3 biological replicates).

### Library preparation, RNA sequencing, and raw data analysis

Total RNA samples were purified through poly(A) + mRNA enrichment and eight directional tagged cDNA libraries were prepared and pooled together. The sequencing was performed on a NextSeq. 500 Illumina platform (Illumina, San Diego, USA) generating approximately 50 million of 75 bp single-end reads per sample. The quality assessment of the sequencing output was performed on FASTQ files using FastQC v0.11.8, the parameters checked were: GC content, per base sequence quality and per sequence quality score (Supplementary Fig. S2, S3, and S4). The raw reads were then aligned to the last Ensembl reference genome available for zebrafish (GRCz11) using HiSat2^45^. The count of the overlapping reads mapped on the reference genome was performed using Htseq-count^46^, which generated the raw count files.

### Determination of the differently expressed genes (DEGs), gene ontology (GO) and pathway enrichment analysis

The differential gene expression analysis on the raw count was performed using the R package Deseq2 ^47^ v.3.8. The results produced by DEseq2 are expressed as the estimated log2 fold change of the unigene expression level between treatments. Each log2 fold change value is coupled with the relative *p*-value, resulting from the statistical test. DEseq2 uses also the Benjamini-Hochberg (BH) adjustment to prevent the multiple comparison errors, returning an adjusted *p*-value which represents the false discovery rate (FDR). The FDR cutoff value to select the differently expressed genes (DEG) was set at 0.05. Gene ontology and pathway enrichment on the DEG list was carried out using DAVID online v.6.8^48,49^ and the pathways available on KEGG (Kyoto Encyclopedia of Genes and Genomes)^50,51^. Benjamini-Hochberg test for the FDR correction was included in the enrichment analysis.

### Primer design and quantitative real-time PCR (qRT-PCR)

A list of DEG was selected from the RNAseq results for qRT-PCR validation. The gene selection was made considering the fold change and corresponding adjusted *p*-value. Primers for the selected genes were obtained from literature or designed with Primer3^52^, on different exons to exclude any genomic DNA co-amplification (Supplementary Table S2). Reverse transcription was performed using iScript™ cDNA synthesis kit (Bio-Rad, California, USA) according to the manufacturer’s instructions using 1 µg total RNA. Prior the qRT-PCR the amplicon length was checked through PCR and agarose gel electrophoresis. The qRT-PCR reaction was performed in triplicates in 96 wells plates, using the SsoAdvanced™ Universal SYBR® Green Supermix (Bio-Rad) on an iCycleriQ5 (Bio-Rad). The amplification efficiency of each primer couple was checked through the creation of a five points standard curve with serially diluted 1:5 cDNA (Supplementary Table S2). The qRT-PCR results were normalized using a set of three house-keeping genes, actin beta 1 (*actb1*), eukaryotic translation elongation factor 1 alpha 1 (*eef1a1*) and ribosomal protein L8 (*rpl8*), which have been identified as stable genes in zebrafish^53–55^.

### Experiment II: behavioral analysis

Adult zebrafish (n = 64) were placed in three 8 L aquaria; 16 fish for the Control (CNTR) group and 24 fish for each treatment, L-MPs and H-MPs doses. After 20 days of exposure, zebrafish were transferred to 8 different recording aquaria (n = 8/aquarium) filled with 8 L of water. Each aquarium was exposed to a 12:12 LD cycle. Illumination was provided by means of white LED light lamps (Superlight Technology Co. Ltd., China). Irradiance was measured with a spectro-radiometer (FieldSpec ASD, Colorado, USA) set at 1.62 E + 18 photons m-2 s-1. The temperature was held constant (26 ± 1 °C) by means of water heaters (50 W, Sera GmbH, Germany) and recorded every 10 minutes with data loggers (Hobo Pendant, Onset Computer Corporation, Massachusetts, USA). Two aquaria were set up for Control groups and other 3 for each treatment. Locomotor activity was recorded for 14 days by an infrared photocell (E3Z-D67; Omron, Kyoto, Japan) placed in each tank. The photocells were connected to a computer, and every time a fish interrupted the infrared light beam it produced an output signal that was recorded and stored in 10-min bins using specialized software (DIO98USB; University of Murcia, Spain). During the recording phase, zebrafish were fed with regular food and 50% of the water was changed three times, on day 3, 8 and 11.

### Statistical analysis and graphics elaboration

Statistical analysis and graphics elaborations on the expression data were performed using a combination of RStudio v.1.1.453 and Genespring GX ver. 14.9. The locomotor activity displayed by zebrafish during the experiment was analyzed (actograms and mean wave-forms) by chronobiology software (El Temps, v.1.179). Kruskal-Wallis one-way ANOVA and Dunn’s Multiple Comparison post-hoc tests were performed using Prism 5.0 (GraphPad Software Inc., USA).

## Supplementary information

Supplementary information

Supplementary information Table S10

## Data Availability

The RNA sequencing data are available online at NCBI sequence read archive (SRA) under the BioProject ID: PRJNA529759 and BioSample accessions: SAMN11282976, SAMN11282977, SAMN11282978, SAMN11282979, SAMN11282980, SAMN11282981, SAMN11282982, SAMN11282983.
