# Supplementary material for: Microplastics induce transcriptional changes, immune response and behavioral alterations in adult zebrafish
Source: Sci Rep. 2019 Oct 31;9:15775. doi: 10.1038/s41598-019-52292-5 (PMC6823372; doi:10.1038/s41598-019-52292-5)
Supplement: Supplementary file 1 — Supplementary information [file 41598_2019_52292_MOESM1_ESM.docx]

**Microplastics induce transcriptional changes, immune response and behavioral alterations in adult zebrafish**

**Giacomo Limonta^1,*^, Annalaura Mancia^2^, Assja Benkhalqui^2^, Cristiano Bertolucci^2^, Luigi Abelli^2^, Maria Cristina Fossi^1^, Cristina Panti^1^**

^1^Department of Physical, Earth and Environmental Sciences, University of Siena, Siena, 53100, Italy.

^2^Department of Life Sciences and Biotechnology, University of Ferrara, Ferrara, 44121, Italy.

*Corresponding author: limonta@student.unisi.it

**SUPPLEMENTARY INFORMATION**

**Table S1.** Summary of biometric data obtained from all fish in each experimental group: Control, L-MPs (100 µg/L), H-MPs (1000 µg/L). TW: Total weight, L: Total length, LW: Liver weight, KW: Kidney weight, SW: spleen weight. In fish marked with * only gills and gastrointestinal tract were dissected (for histopathological analysis).

| Treatment replicates | Fish | Sex | TW (mg) | L (cm) | LW (mg) | KW (mg) | SW (mg) |
| --- | --- | --- | --- | --- | --- | --- | --- |
| Control | C1 | F | 792 | 3,5 | 17 | 11,9 | <LOD |
|  | C2 | M | 561 | 3,3 | 6,5 | 4,8 | 1,1 |
|  | C3 | F | 678 | 3,4 | 11,7 | 3,3 | 2,2 |
|  | C4* | M | 539 | 3,2 | ꟷ | ꟷ | ꟷ |
| Control | C5 | M | 459 | 3,2 | 17,5 | 2,9 | 1,0 |
|  | C6 | M | 540 | 3,4 | 7,7 | 1,6 | <LOD |
|  | C7 | M | 502 | 3,5 | 6,6 | 1,8 | 0,6 |
|  | C8* | F | 625 | 3,5 | ꟷ | ꟷ | ꟷ |
| Control | C9 | F | 677 | 3,4 | 17,6 | 4,6 | 0,7 |
|  | C10 | M | 380 | 3 | 13,7 | 2,2 | <LOD |
|  | C11 | M | 513 | 3,3 | 13,7 | 2,5 | <LOD |
|  | C12* | M | 384 | 3,1 | ꟷ | ꟷ | ꟷ |
| L-MPs | L1 | F | 710 | 3,7 | 12,3 | 2,6 | <LOD |
|  | L2 | F | 578 | 3 | 18,9 | 2,8 | <LOD |
|  | L3 | M | 588 | 3,6 | 6 | 3,8 | 0,9 |
|  | L4* | M | 511 | 3,4 | ꟷ | ꟷ | ꟷ |
| L-MPs | L5 | M | 598 | 3,3 | 9,7 | 2,7 | <LOD |
|  | L6 | M | 540 | 3,4 | 9,1 | 2,9 | <LOD |
|  | L7 | F | 720 | 3,4 | 19,2 | 4,1 | 1 |
|  | L8* | F | 517 | 3,2 | ꟷ | ꟷ | ꟷ |
| L-MPs | L9 | F | 694 | 3,3 | 23,3 | 3 | 2,1 |
|  | L10 | M | 620 | 3,5 | 23,6 | 3,4 | <LOD |
|  | L11 | F | 792 | 3,8 | 25,3 | 2,6 | <LOD |
|  | L12* | ꟷ | ꟷ | ꟷ | ꟷ | ꟷ | ꟷ |
| H-MPs | H1 | M | 518 | 3,4 | 7,7 | 1,2 | 1,9 |
|  | H2 | F | 576 | 3,3 | 10,6 | 3 | 1,6 |
|  | H3 | M | 479 | 3,5 | 6,1 | 2,3 | 1,6 |
|  | H4* | F | 639 | 3,2 | ꟷ | ꟷ | ꟷ |
| H-MPs | H5 | F | 520 | 3,3 | 18,4 | 2,1 | 1,4 |
|  | H6 | F | 715 | 3,4 | 9,8 | 5,1 | 0,6 |
|  | H7 | M | 596 | 3,7 | 8 | 1,8 | 1,5 |
|  | H8* | F | 802 | 3,4 | ꟷ | ꟷ | ꟷ |
| H-MPs | H9 | F | 667 | 3,4 | 29 | 4,6 | 0,8 |
|  | H10 | F | 633 | 3,5 | 28,4 | 4,7 | 0,8 |
|  | H11 | F | 637 | 3,4 | 21,8 | 2,7 | 0,6 |
|  | H12* | F | 344 | 2,9 | ꟷ | ꟷ | ꟷ |

**Table S2.** Primers used for genes of interest (GOIs) and housekeeping genes (HKs) in the RT-qPCR validation of the RNAseq results.

|  | Gene symbol | Reference sequence | Primer sequence | | Amplicon size (bp) | Primer efficiency | r^2^ |
| --- | --- | --- | --- | --- | --- | --- | --- |
| GOIs | *cyp5* | NM_001001730.2 | Fw  Rv | TTCGCCTACATCCCATTCG  AACATCCGCAGCAGAGTC | 101 | 97,5% | 0,995 |
|  | *ifitm1* | NM_001110287.1 | Fw  Rv | GATGACAAAACCTGCAACGG  GATGTTCAGACGTCGAGCTC | 219 | 94,9% | 0,999 |
|  | *ch25h*^1^ | NM_001008652.1 | Fw  Rv | CGGTGAATCCCATGTTGCTT  AGCTCCTCCGTAAAGTCCAAAA | 149 | 90% | 0,993 |
|  | *elovl6* | NM_199532.1 | Fw  Rv | TTCCTCTTCTCTGCGCTCTA  AACTCTGATCGCACACTGAC | 208 | 107,5% | 0,995 |
|  | *ltb4r* | NM_001308975.1 | Fw  Rv | CCATGCCATTTTATCGCTCT  AGAACAGGATGAAGGCGAAG | 156 | 93,8% | 0,977 |
| HKs | *actb1* | NM_131031.1 | Fw  Rv | ATCCACGAGACCACCTTCAA  GTACTCCTGCTTGCTGATCC | 267 | 103,3% | 0,997 |
|  | *eef1a1* | NM_131263.1 | Fw  Rv | GGAAGTCAGCGCATACATCA  CGCTAGCATTACCCTCCTTG | 158 | 94% | 0,998 |
|  | *rpl8*^2^ | NM_200713.1 | Fw  Rv | ATGTTTCGTGACCCTTACCG  CTCCTCCAGACAGCAGACTA | 171 | 100,6% | 0,998 |

**Table S3.** GO terms significantly enriched (*p<0,05*) after H-MPs treatment (1000 µg/L).

| Category | GO Term | | Count | % | PValue | Fold Enrichment | FDR |
| --- | --- | --- | --- | --- | --- | --- | --- |
| Biological | **GO:0008152** | metabolic process | 11 | 6.32 | 3.9E-04 | 3.99 | 0.08 |
| process | **GO:0043691** | reverse cholesterol transport | 3 | 1.72 | 2.2E-03 | 41.50 | 0.22 |
|  | **GO:0033344** | cholesterol efflux | 3 | 1.72 | 4.7E-03 | 28.53 | 0.30 |
|  | **GO:0016126** | sterol biosynthetic process | 3 | 1.72 | 5.3E-03 | 26.85 | 0.26 |
|  | **GO:0006629** | lipid metabolic process | 6 | 3.45 | 8.0E-03 | 4.81 | 0.30 |
|  | **GO:0008202** | steroid metabolic process | 3 | 1.72 | 9.7E-03 | 19.85 | 0.31 |
|  | **GO:0008203** | cholesterol metabolic process | 3 | 1.72 | 1.3E-02 | 16.91 | 0.35 |
|  | **GO:0006633** | fatty acid biosynthetic process | 3 | 1.72 | 3.0E-02 | 10.87 | 0.58 |
| Cellular | **GO:0016021** | integral component of membrane | 57 | 32.76 | 1.5E-02 | 1.28 | 0.56 |
| component | **GO:0005783** | endoplasmic reticulum | 8 | 4.60 | 2.4E-02 | 2.78 | 0.50 |

**Table S4.** GO terms significantly enriched (*p<0,05*) after L-MPs treatment (100 µg/L).

| Category | GO Term | | Count | % | PValue | Fold Enrichment | FDR |
| --- | --- | --- | --- | --- | --- | --- | --- |
| Biological | **GO:0016126** | sterol biosynthetic process | 8 | 3.70 | 1.15E-10 | 48.73 | 3.78E-08 |
| preocess | **GO:0006695** | cholesterol biosynthetic process | 7 | 3.24 | 1.97E-09 | 51.78 | 3.23E-07 |
|  | **GO:0008299** | isoprenoid biosynthetic process | 6 | 2.78 | 5.67E-08 | 51.78 | 6.18E-06 |
|  | **GO:0008202** | steroid metabolic process | 7 | 3.24 | 6.18E-08 | 31.52 | 5.05E-06 |
|  | **GO:0006694** | steroid biosynthetic process | 7 | 3.24 | 8.17E-08 | 30.20 | 5.34E-06 |
|  | **GO:0008203** | cholesterol metabolic process | 7 | 3.24 | 1.76E-07 | 26.85 | 9.57E-06 |
|  | **GO:0006629** | lipid metabolic process | 11 | 5.09 | 1.47E-05 | 6.00 | 6.85E-04 |
|  | **GO:0019287** | isopentenyl diphosphate biosynthetic process | 3 | 1.39 | 2.72E-04 | 103.56 | 0.011068 |
|  | **GO:0055114** | oxidation-reduction process | 16 | 7.41 | 8.44E-04 | 2.69 | 0.030221 |
|  | **GO:0043691** | reverse cholesterol transport | 3 | 1.39 | 4.75E-03 | 28.24 | 0.144058 |
|  | **GO:0001558** | regulation of cell growth | 4 | 1.85 | 6.13E-03 | 10.62 | 0.167089 |
|  | **GO:0006633** | fatty acid biosynthetic process | 4 | 1.85 | 7.54E-03 | 9.86 | 0.18643 |
|  | **GO:0033344** | cholesterol efflux | 3 | 1.39 | 1.00E-02 | 19.42 | 0.224061 |
|  | **GO:0006954** | inflammatory response | 6 | 2.78 | 1.16E-02 | 4.41 | 0.237683 |
|  | **GO:0048884** | neuromast development | 3 | 1.39 | 1.70E-02 | 14.79 | 0.312186 |
|  | **GO:0008152** | metabolic process | 10 | 4.63 | 1.97E-02 | 2.47 | 0.333622 |
|  | **GO:0001878** | response to yeast | 3 | 1.39 | 2.20E-02 | 12.94 | 0.347552 |
|  | **GO:0051216** | cartilage development | 4 | 1.85 | 4.29E-02 | 5.11 | 0.548771 |
|  | **GO:0030593** | neutrophil chemotaxis | 3 | 1.39 | 4.65E-02 | 8.63 | 0.559558 |
| Cellular | **GO:0005783** | endoplasmic reticulum | 17 | 7.87 | 1.53E-06 | 4.39 | 1.13E-04 |
| component | **GO:0005789** | endoplasmic reticulum membrane | 13 | 6.02 | 1.23E-05 | 4.95 | 4.55E-04 |
|  | **GO:0005576** | extracellular region | 16 | 7.41 | 3.70E-03 | 2.31 | 0.087417 |
|  | **GO:0016020** | membrane | 74 | 34.26 | 1.81E-02 | 1.23 | 0.286354 |
|  | **GO:0016021** | integral component of membrane | 73 | 33.80 | 2.42E-02 | 1.21 | 0.304314 |
| Molecular | **GO:0016491** | oxidoreductase activity | 16 | 7.41 | 3.12E-04 | 2.97 | 0.088524 |
| function | **GO:0071949** | FAD binding | 3 | 1.39 | 1.43E-02 | 16.20 | 0.882305 |
|  | **GO:0042289** | MHC class II protein binding | 2 | 0.93 | 1.93E-02 | 102.58 | 0.854432 |
|  | **GO:0005520** | insulin-like growth factor binding | 3 | 1.39 | 3.61E-02 | 9.93 | 0.934655 |

**Table S5.** KEGG patways significantly enriched (*p<0,05*) after H-MPs treatment (1000 µg/L).

| Category | Term | | Count | % | PValue | Fold Enrichment | FDR |
| --- | --- | --- | --- | --- | --- | --- | --- |
| KEGG | **dre00072** | Synthesis and degradation of ketone bodies | 3 | 1.72 | 0.002351 | 39.16667 | 0.131723 |
| pathway | **dre01130** | Biosynthesis of antibiotics | 7 | 4.02 | 0.011967 | 3.530043 | 0.303144 |
|  | **dre00100** | Steroid biosynthesis | 3 | 1.72 | 0.012873 | 16.78571 | 0.228282 |
|  | **dre00900** | Terpenoid backbone biosynthesis | 3 | 1.72 | 0.014086 | 16.02273 | 0.191679 |
|  | **dre00650** | Butanoate metabolism | 3 | 1.72 | 0.018007 | 14.1 | 0.195921 |
|  | **dre03320** | PPAR signaling pathway | 4 | 2.30 | 0.018546 | 6.911765 | 0.170722 |
|  | **dre00280** | Valine, leucine and isoleucine degradation | 3 | 1.72 | 0.06914 | 6.778846 | 0.458878 |
|  | **dre01212** | Fatty acid metabolism | 3 | 1.72 | 0.083561 | 6.077586 | 0.48027 |

**Table S6.** KEGG patways significantly enriched (*p<0,05*) after L-MPs treatment (100 µg/L).

| Category | Term | | Count | % | PValue | Fold Enrichment | FDR |
| --- | --- | --- | --- | --- | --- | --- | --- |
| KEGG | **dre00100** | Steroid biosynthesis | 9 | 4.17 | 1.65E-10 | 30.47932 | 1.11E-08 |
| pathway | **dre01130** | Biosynthesis of antibiotics | 19 | 8.80 | 1.47E-09 | 5.799356 | 4.92E-08 |
|  | **dre00900** | Terpenoid backbone biosynthesis | 8 | 3.70 | 1.08E-08 | 25.86124 | 2.40E-07 |
|  | **dre01100** | Metabolic pathways | 29 | 13.42 | 0.00401 | 1.642065 | 0.065088 |
|  | **dre04146** | Peroxisome | 5 | 2.31 | 0.035672 | 3.951023 | 0.385371 |
|  | **dre04141** | Protein processing in endoplasmic reticulum | 7 | 3.24 | 0.038814 | 2.750436 | 0.357284 |
|  | **dre00650** | Butanoate metabolism | 3 | 1.39 | 0.046402 | 8.534211 | 0.365406 |

**Table S7.** DEGs having a function correlated with the immune system. The colored box next to the gene description indicates in which MPs treatment the gene was found differentially expressed compared to the control. Yellow: differently expressed in both L-MPs and H-MPs. Green: differently expressed in L-MPs. Blue: differently expressed in H-MPs

| **Up-regulated** |  |  |  |
| --- | --- | --- | --- |
| **Ensemble gene ID** | **Gene symbol** | **Description** |  |
| ENSDARG00000009087 | *cd74a* | major histocompatibility complex, class II invariant chain a |  |
| ENSDARG00000012539 | *dnase1* | deoxyribonuclease I |  |
| ENSDARG00000016835 | *tcirg1a* | T cell immune regulator 1, ATPase H+ transporting V0 subunit a3a |  |
| ENSDARG00000035632 | *ccl19a.2* | chemokine (C-C motif) ligand 19a, tandem duplicate 2 |  |
| ENSDARG00000036628 | *cd74b* | major histocompatibility complex, class II invariant chain b |  |
| ENSDARG00000039351 | *ccl19b* | chemokine (C-C motif) ligand 19b |  |
| ENSDARG00000041923 | *ccl38.6* | chemokine (C-C motif) ligand 38, duplicate 6 |  |
| ENSDARG00000044561 | *ccr7* | chemokine (C-C motif) receptor 7 |  |
| ENSDARG00000052437 | *mia* | MIA SH3 domain containing |  |
| ENSDARG00000079773 | *nox5* | NADPH oxidase, EF-hand calcium binding domain 5 |  |
| ENSDARG00000086483 | *LECT2* | si:ch211-132p1.4 |  |
| ENSDARG00000090851 | *ciita* | class II, major histocompatibility complex, transactivator |  |
| ENSDARG00000098365 | *lrrc74b* | leucine rich repeat containing 74B |  |
| ENSDARG00000099416 | *ly6m3* | lymphocyte antigen 6 family member M3 |  |
| ENSDARG00000099684 | *trim35-24* | tripartite motif containing 35-24 |  |
| ENSDARG00000101055 | *trim35-25* | tripartite motif containing 35-25 |  |
| ENSDARG00000102368 | *trim35-23* | tripartite motif containing 35-23 |  |
| **Down-regulated** |  |  |  |
| **Ensemble gene ID** | **Gene symbol** | **Description** |  |
| ENSDARG00000002235 | *mmp14a* | matrix metallopeptidase 14a (membrane-inserted) |  |
| ENSDARG00000003061 | *cd276* | CD276 molecule |  |
| ENSDARG00000003570 | *hsp90b1* | heat shock protein 90, beta (grp94), member 1 |  |
| ENSDARG00000006990 | *creld1b* | cysteine-rich with EGF-like domains 1b |  |
| ENSDARG00000008388 | *mmp14b* | matrix metallopeptidase 14b (membrane-inserted) |  |
| ENSDARG00000018404 | *krt18* | keratin 18a, tandem duplicate 1 |  |
| ENSDARG00000019815 | *fn1a* | fibronectin 1a |  |
| ENSDARG00000025670 | *fstl3* | follistatin-like 3 (secreted glycoprotein) |  |
| ENSDARG00000032631 | *ltb4r* | leukotriene B4 receptor |  |
| ENSDARG00000037962 | *psmb7* | proteasome subunit beta 7 |  |
| ENSDARG00000042577 | *batf3* | basic leucine zipper transcription factor, ATF-like 3 |  |
| ENSDARG00000045748 | *stab2* | stabilin 2 |  |
| ENSDARG00000052322 | *tlr5b* | toll-like receptor 5b |  |
| ENSDARG00000057173 | *ifit8* | interferon-induced protein with tetratricopeptide repeats 8 |  |
| ENSDARG00000059294 | *marco* | macrophage receptor with collagenous structure |  |
| ENSDARG00000059327 | *gata2a* | GATA binding protein 2a |  |
| ENSDARG00000062552 | *lpar6a* | lysophosphatidic acid receptor 6a |  |
| ENSDARG00000062707 | *plat* | plasminogen activator, tissue |  |
| ENSDARG00000070012 | *sesn2* | sestrin 2 |  |
| ENSDARG00000070669 | *cxcr3.3* | chemokine (C-X-C motif) receptor 3, tandem duplicate 3 |  |
| ENSDARG00000074851 | *s1pr4* | sphingosine-1-phosphate receptor 4 |  |
| ENSDARG00000075445 | *psmb5* | proteasome subunit beta 5 |  |
| ENSDARG00000089131 | *il17rel* | interleukin 17 receptor E-like |  |
| ENSDARG00000093303 | *ifitm1* | interferon induced transmembrane protein 1 |  |
| ENSDARG00000104795 | *cxcl8a* | chemokine (C-X-C motif) ligand 8a |  |
| ENSDARG00000111526 | fam20a | pseudokinase FAM20A-like |  |

**Table S8**. DEGs correlated with lipid metabolism. The colored box next to the gene description indicates in which MPs treatment the gene was found differentially expressed compared to the control. Yellow: differently expressed in both L-MPs and H-MPs. Green: differently expressed in L-MPs. Blue: differently expressed in H-MPs

| **Down-regulated** |  |  |  |
| --- | --- | --- | --- |
| **Ensemble gene ID** | **Gene symbol** | **Description** |  |
| ENSDARG00000004130 | *mvk* | mevalonate kinase |  |
| ENSDARG00000004402 | *elovl6* | ELOVL fatty acid elongase 6 |  |
| ENSDARG00000012468 | *aacs* | acetoacetyl-CoA synthetase |  |
| ENSDARG00000019976 | *idi1* | isopentenyl-diphosphate delta isomerase 1 |  |
| ENSDARG00000031044 | *lipg* | lipase, endothelial |  |
| ENSDARG00000040295 | *apoeb* | apolipoprotein Eb |  |
| ENSDARG00000040890 | *fdps* | farnesyl diphosphate synthase) |  |
| ENSDARG00000045190 | *ch25h* | cholesterol 25-hydroxylase |  |
| ENSDARG00000052734 | *hmgcra* | 3-hydroxy-3-methylglutaryl-CoA reductase a |  |
| ENSDARG00000053010 | *lpcat2* | lysophosphatidylcholine acyltransferase 2 |  |
| ENSDARG00000055876 | *msmo1* | methylsterol monooxygenase 1 |  |
| ENSDARG00000063438 | *srebf2* | sterol regulatory element binding transcription factor 2 |  |
| ENSDARG00000070798 | *pmvk* | phosphomevalonate kinase |  |
| ENSDARG00000079009 | *abca1b* | ATP-binding cassette, sub-family A (ABC1), member 1B |  |
| ENSDARG00000079946 | *sqlea* | squalene epoxidase a |  |
| ENSDARG00000095863 | *afp4* | antifreeze protein type IV |  |
| ENSDARG00000099315 | *nsdhl* | NAD(P) dependent steroid dehydrogenase-like |  |
| ENSDARG00000099336 | *mvda* | mevalonate (diphospho) decarboxylase a |  |
| ENSDARG00000099424 | *lcat* | lecithin-cholesterol acyltransferase |  |
| ENSDARG00000101062 | *fdft1* | farnesyl-diphosphate farnesyltransferase 1 |  |
| ENSDARG00000103025 | *hmgcs1* | 3-hydroxy-3-methylglutaryl-CoA synthase 1 (soluble) |  |
| ENSDARG00000103226 | *dhcr7* | 7-dehydrocholesterol reductase |  |
| ENSDARG00000020149 | *acoxl* | acyl-CoA oxidase-like |  |

**Table S9.** DEGs correlated with cellular proliferation and apoptosis. The colored box next to the gene description indicates in which MPs treatment the gene was found differentially expressed compared to the control. Yellow: differently expressed in both L-MPs and H-MPs. Green: differently expressed in L-MPs. Blue: differently expressed in H-MPs

| **Up-regulated** |  |  |  |
| --- | --- | --- | --- |
| **Ensemble gene ID** | **Gene symbol** | **Description** |  |
| ENSDARG00000076858 | *TCIM* | transcriptional and immune response regulator b |  |
| **Down-regulated** |  |  |  |
| **Ensemble gene ID** | **Gene symbol** | **Description** |  |
| ENSDARG00000028017 | *tp53inp1* | tumor protein p53 inducible nuclear protein 1 |  |
| ENSDARG00000007614 | *wwox* | WW domain containing oxidoreductase |  |
| ENSDARG00000070480 | *agr2* | anterior gradient 2 |  |
| ENSDARG00000058992 | *cers2b* | ceramide synthase 2b |  |

**
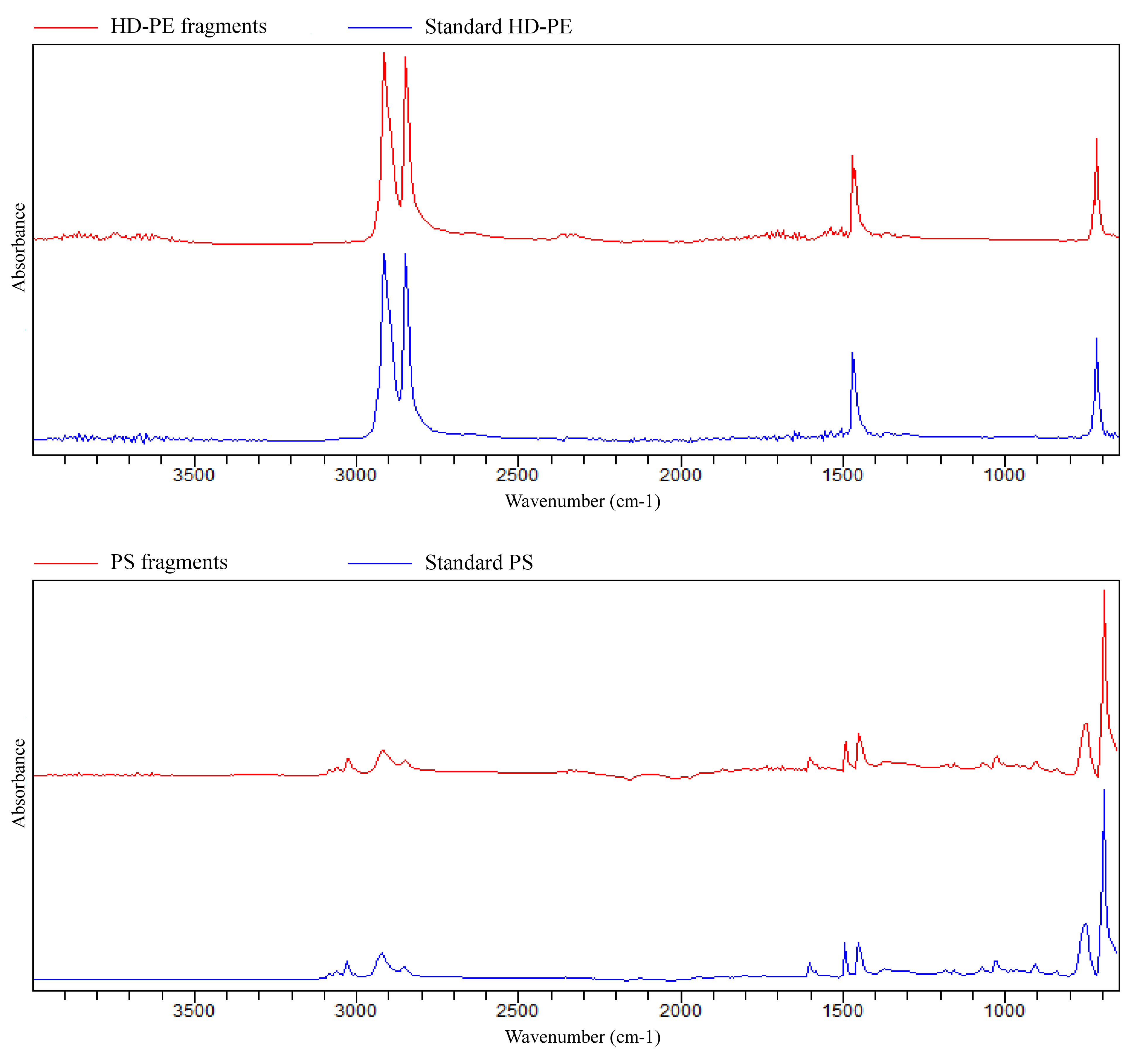
**

**Figure S1.** FTIR spectrum of HD-PE (high-density polyethylene) and PS (polystyrene) fragments used in the experiment and the standard spectrum for HD-PE and PS present in the instrument library (Agilent Polymer ATR Library). Instrument: Cary 630 FTIR spectrometer (Agilent); The spectrum were acquired using the FTIR software MicroLab v.5.2.1612.0. sample scans: 256; background scans: 128; resolution: 4; apodization: Happ-Genzel.

**
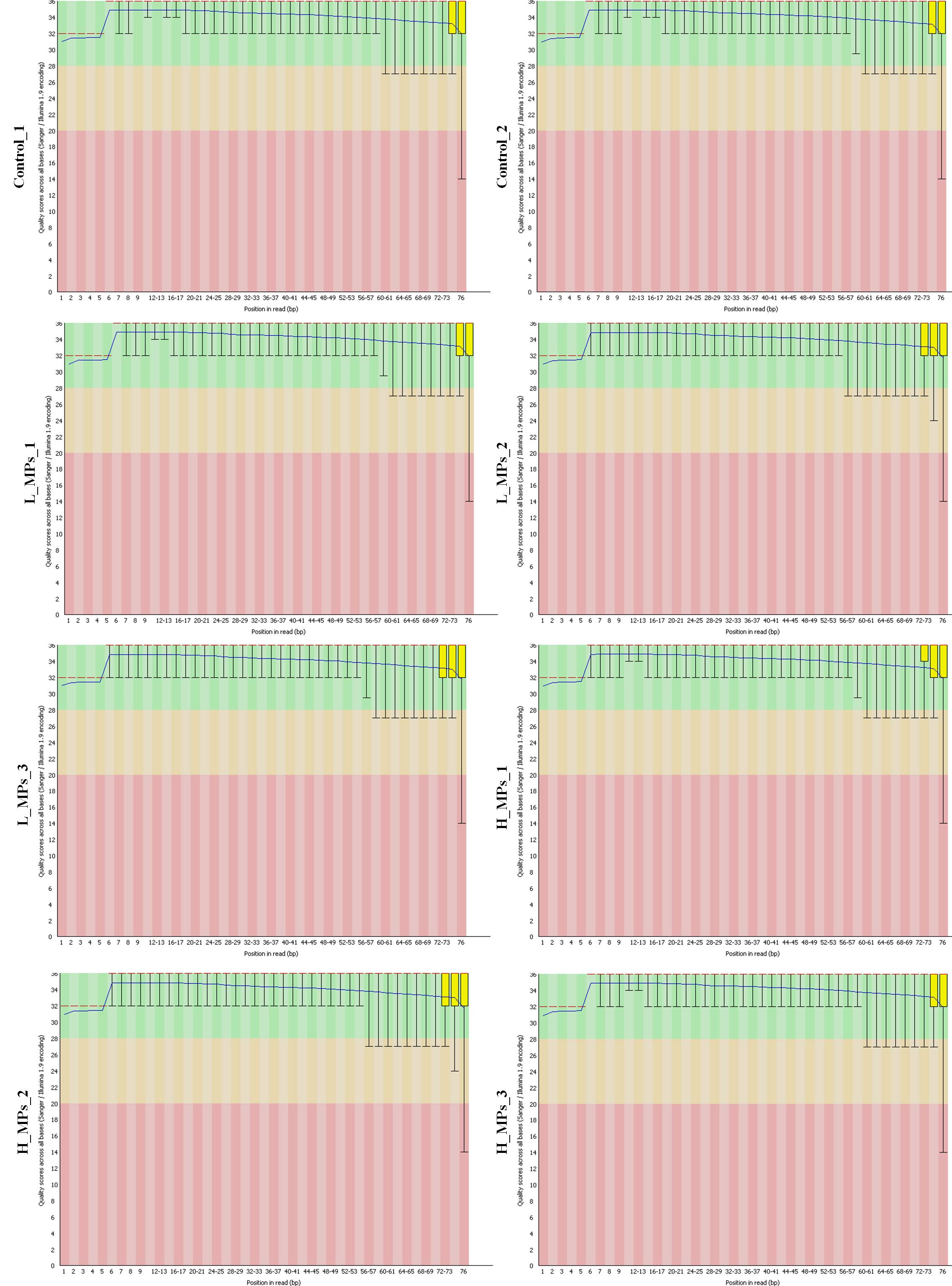
**

**Figure S2.** Average quality score per base obtained for the results of RNA sequencing for all of the samples.


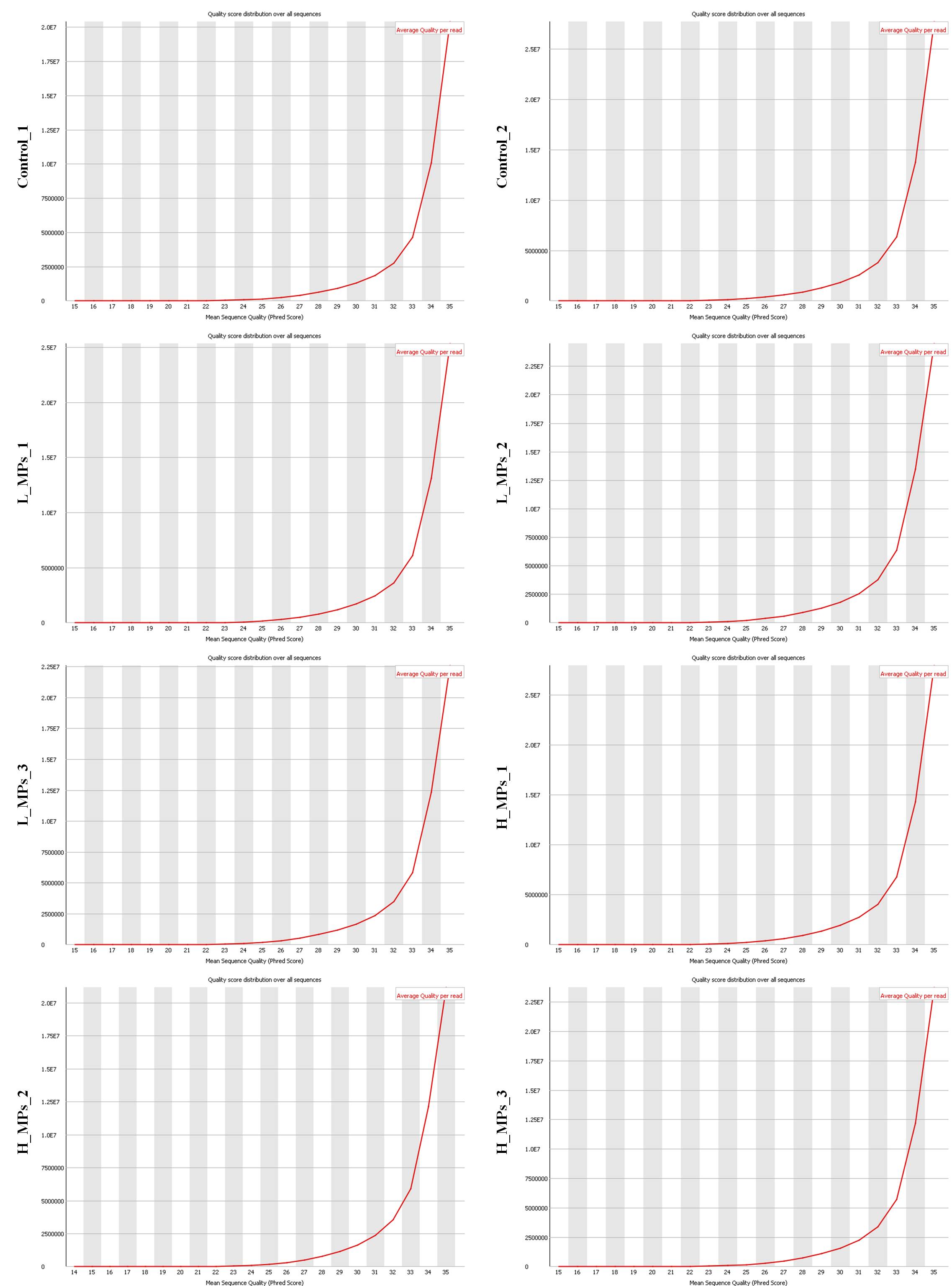


**Figure S3.** Average quality score per sequence obtained for the results of RNA sequencing all of the samples.

**
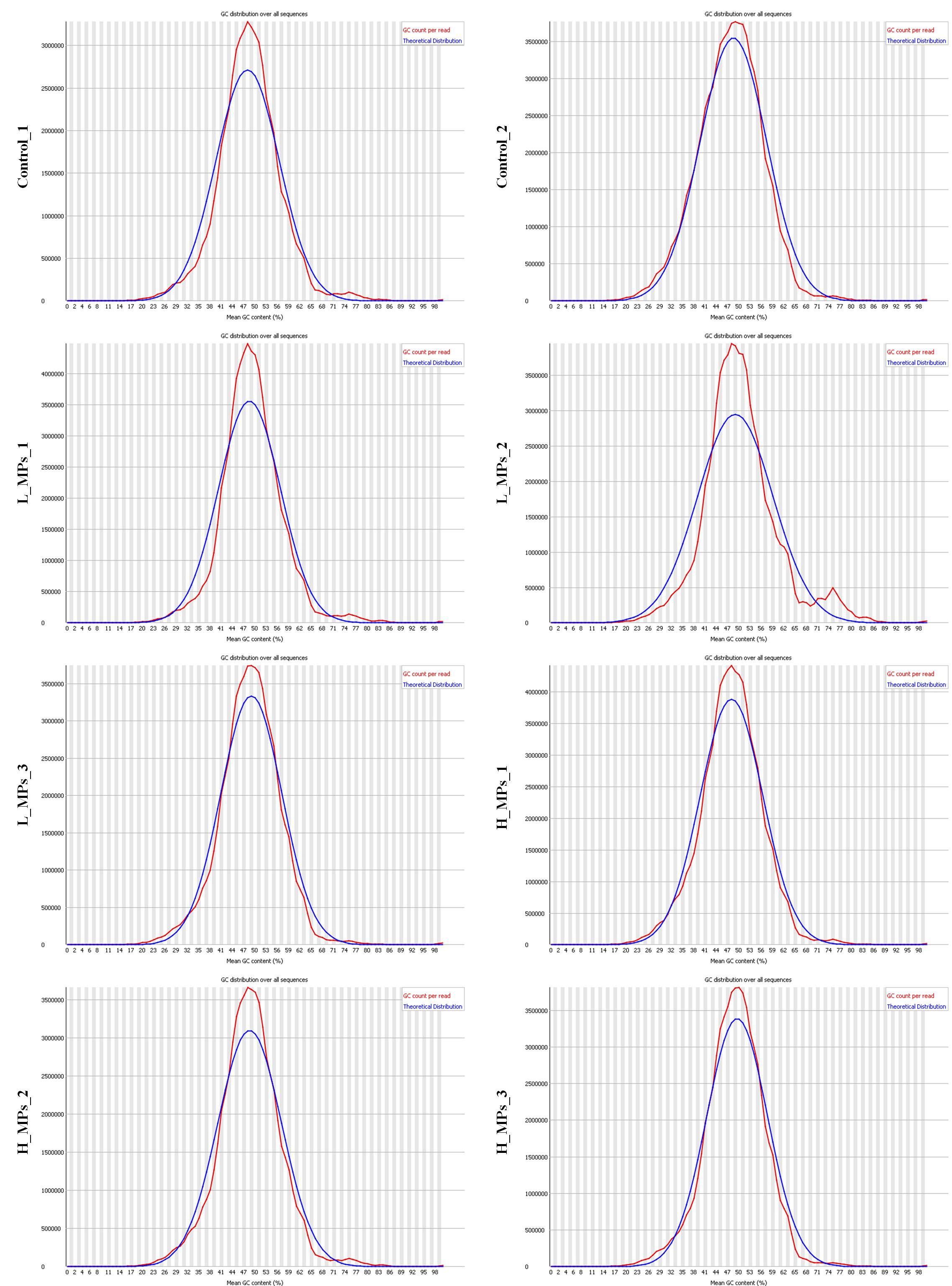
**

**Figure S4.** Average GC content of all the sequences obtained from the RNA sequencing for all of the samples.

**
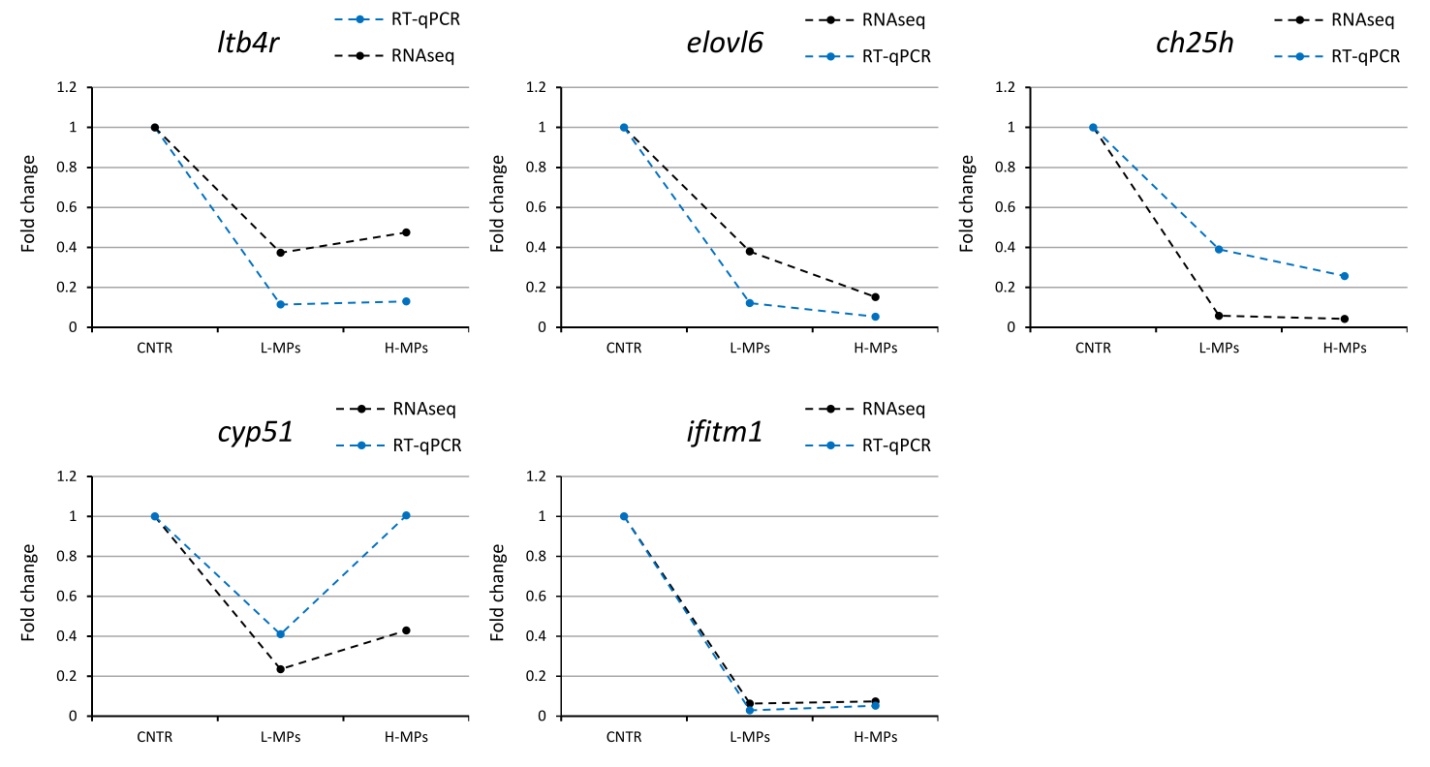
**

**Figure S5.** Validation of RNAseq data through qRT-PCR. Comparison between the fold change of expression obtained through RNAseq and qRT-PCR for the selected genes. The fold change of expression for each gene in both MPs treatments, L-MPs (100 µg/L); H-MPs (1000 µg/L), is relative to CNRT (control) which is set at 1.

**References**

1. Dios, S. *et al.* The Involvement of Cholesterol in Sepsis and Tolerance to Lipopolysaccharide Highlighted by the Transcriptome Analysis of Zebrafish ( *Danio rerio* ). *Zebrafish* **11**, 421–433 (2014).

2. Brown, A. R. *et al.* Are Toxicological Responses in Laboratory (Inbred) Zebrafish Representative of Those in Outbred (Wild) Populations? − A Case Study with an Endocrine Disrupting Chemical. *Environmental Science & Technology* **45**, 4166–4172 (2011).
